# A Temperature-Insensitive Cladding-Etched Fiber Bragg Grating Using a Liquid Mixture with a Negative Thermo-Optic Coefficient

**DOI:** 10.3390/s120607886

**Published:** 2012-06-08

**Authors:** Kwang Taek Kim, In Soo Kim, Cherl-Hee Lee, Jonghun Lee

**Affiliations:** 1 Department of Optoelectronics, Honam University, Gwangju 506-714, Korea; 2 Robot System Division, Daegu Gyeongbuk Institute of Science & Technology, Daegu 711-813, Korea

**Keywords:** temperature insensitive, cladding etched, Fiber Bragg grating, compensation, negative thermo-optic coefficient

## Abstract

To compensate for the temperature dependency of a standard FBG, a cladding-etched FBG immersed with a liquid mixture having a negative thermo-optic coefficient is presented, and its characteristics are investigated. The Bragg wavelength of the cladding-etched FBG is shifted counter to the direction of the Bragg wavelength shift of a conventional FBG according to the mixing ratio of glycerin to water; thus, the temperature-dependent Bragg wavelength shift was almost compensated by using a liquid mixture of water (50%) and glycerin (50%) having the negative thermo-optic coefficient of −5 × 10^−4^ °C^−1^.

## 1. Introduction

Fiber Bragg gratings (FBGs) are receiving much attention for fiber sensor applications due to their small size, absolute measurement capability, immunity to electromagnetic interference, wavelength multiplexing, and distributed sensing possibilities. Since they are readily made by controlling the period, length, amplitude, apodization, and chirp of a fiber grating, FBGs have been extensively studied as optical fiber sensors for measuring temperature [1], strain [2], pressure [3], acceleration [4], torsion [5], flow [6], *etc*. FBG sensors offer high sensitivity, real-time processing, and long-term stability, as well as other important advantages.

However, due to the thermo-optic coefficient of silica and its thermal expansion coefficient, FBG sensors have a temperature-dependent Bragg wavelength shift of 13.7 pm/°C [7]; therefore, the ability to eliminate the thermal effect remains a challenge, and considerable research has been carried out to compensate the temperature dependence by using two gratings [8] and interrogation at two separate wavelengths [9], and bending effects [10]. These designs require additional components or the sensors must be positioned in a suitable geometrical structure to compensate for the temperature effect. Coating the cladding of an FBG with temperature-sensitive materials has also been found to have a significant effect on thermal sensitivity [11]. In [11], the large thermal expansion of the coating-polymer induces an axial strain due to thermal stresses and changes the refractive index of the fiber core and fiber length, thereby improving the thermal sensitivity of the FBG. It is well known that the temperature dependence of the refractive index of an optical fiber core makes the Bragg wavelength shift to a longer wavelength with increasing temperature.

Recently, an etched FBG has been investigated for the measurement of refractive indexes using the thermo-optic coefficient of an external liquid [12,13]. It is widely accepted that the etched cladding of an FBG undergoes a strong mode-coupling with surrounding materials, leading to a strong change of the effective refractive index of an FBG. Here, if a coating material with a negative thermo-optic coefficient is used on a cladding-etched FBG, the effective refractive index of the cladding-etched FBG can be lower, and as a result, the temperature dependence of the FBG can be significantly diminished.

**Figure 1.**
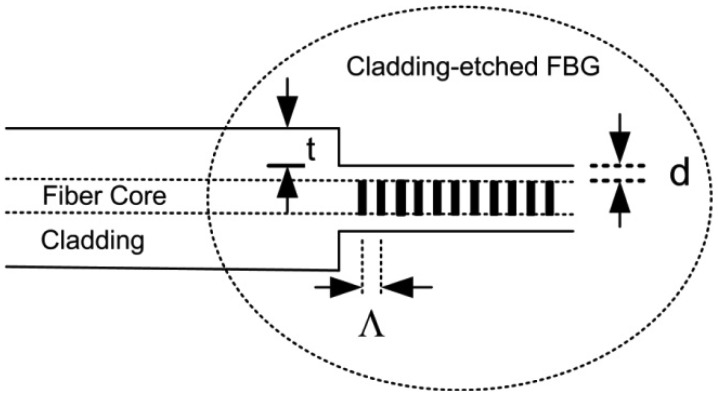
Structure of the cladding-etched FBG.

In this paper, a new temperature-compensation method is presented and experimentally demonstrated in which a liquid mixture with a negative thermo-optic coefficient is used as an external coating material of a cladding-etched FBG. Figure 1 shows the structure of the fabricated cladding-etched FBG etched almost to the fiber core to get the 0.3-μm-radius remained cladding (d), 61.2-μm-thickness removed cladding (t), and grating pitch of 535 nm (Λ).

## 2. Experiments

An FBG is a type of distributed Bragg reflector having a periodic variation in the refractive index of an optical fiber core, which is fabricated by exposing a photosensitized fiber core to ultraviolet light. The reflected wavelength, the Bragg wavelength, is defined as:

(1) $$\lambda_{B}=2n_{\text{eff}}\Lambda .$$

**Figure 2.**
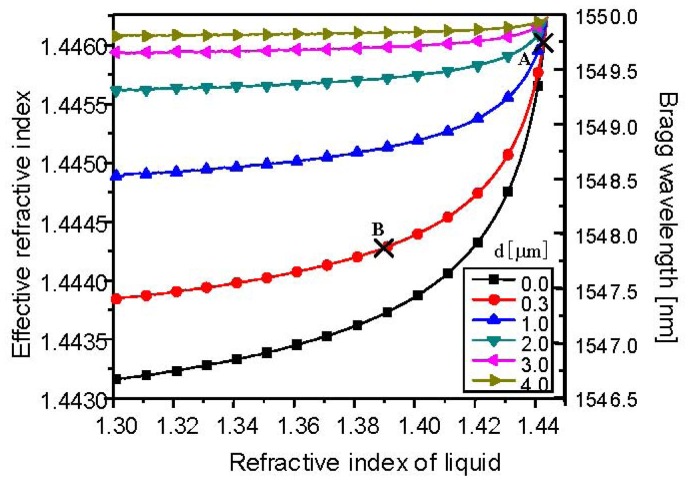
Effective refractive index and Bragg wavelength of cladding-etched single-mode FBG in relation to the refractive index of the external medium for various remaining-cladding thicknesses.

Generally, the grating period (Λ) and effective refractive index (n_eff_) of a single-mode fiber core have a thermal response to the temperature applied to the fiber core. In the case of silica fibers, the thermal response is dominated by the refractive index change rather than the thermal expansion of the fiber core, accounting for more than 95% of the Bragg wavelength shift [14]. As a result of the change of refractive index, the Bragg wavelength shifts to the longer wavelength with a temperature sensitivity of 0.01 nm/°C. In addition, the Bragg wavelength of the cladding-etched FBG also depends on the refractive index of the external medium because the fiber mode profile and its effective refractive index are affected by evanescent wave coupling. Figure 2 shows the effective refractive index of the cladding-etched single-mode FBG in relation to the refractive index of a liquid as an external medium (n_ex_) for several remaining-cladding thicknesses (*d*).

The eigenvalues were calculated by using the doubly clad theory [15] for the cladding-etched single-mode fiber that consisted of a fiber core, an etched cladding, and a liquid as an outer cladding. A standard single-mode optical fiber (SMF 28) with a cladding diameter of 125 μm, core diameter of 8.2 μm, and 0.36% relative refractive index difference was considered in the calculation. The refractive indices of the core and cladding were 1.449 and 1.444, respectively.

As shown in Figure 2, the effective refractive index of the fiber core decreases as the refractive index of the liquid as an external medium decreases. As the remaining-cladding thickness becomes smaller, the effective refractive index of the fiber core decreases more for a fixed refractive index of the liquid. When *d* = 0.3 μm and the refractive index of the liquid is 1.44, point A has the Bragg wavelength of 1,549.8 nm, which shifts to a shorter wavelength of 1,547.8 nm convertible to a Bragg wavelength shift of −2 nm by adjusting the refractive index of the liquid to 1.385 (point B). A properly designed liquid with a negative thermo-optic coefficient, the same magnitude with a positive thermo-optic coefficient of the fiber core, can counteract the temperature-dependent shifts of the Bragg wavelength of the silica fiber. Thus, this method directly compensates the thermal fluctuation of a Bragg wavelength of a FBG.

**Figure 3.**
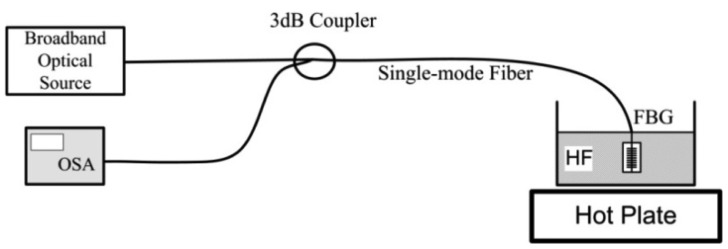
Experimental setup for cladding-etching process.

Figure 3 shows the experimental setup for the cladding-etching process in which the spectrum is monitored during and after the etching of an FBG connected to the 3 dB coupler. A broadband optical source (Agilent 83437A) and an optical spectrum analyzer (OSA, Ando, AQ6315A) were used to measure the reflected optical power through the 3 dB coupler. The FBG fiber was immersed and chemically etched in an aqueous solution of hydrofluoric acid (HF 40%) at 60 °C, and the Bragg wavelength shift relative to the initial Bragg wavelength was monitored in real time during the chemical etching process. With the approximate etching rate of 1.1 μm/min, the fiber cladding was etched almost to the fiber core for evanescent wave coupling with an external medium.

**Figure 4.**
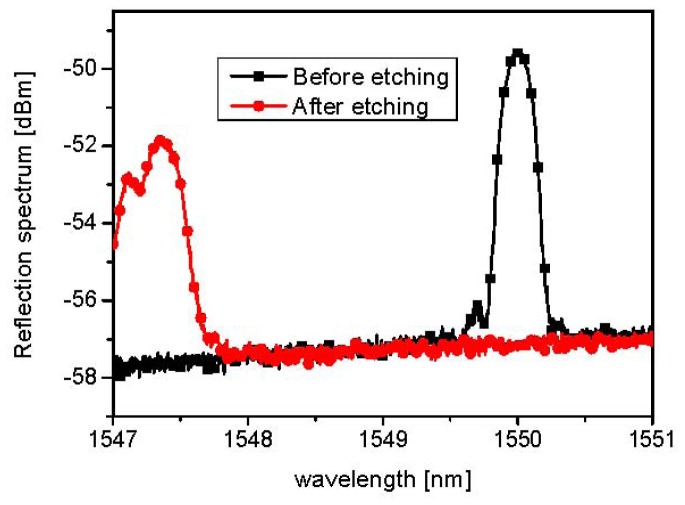
Measured Bragg wavelength before and after the cladding-etching process.

**Figure 5.**
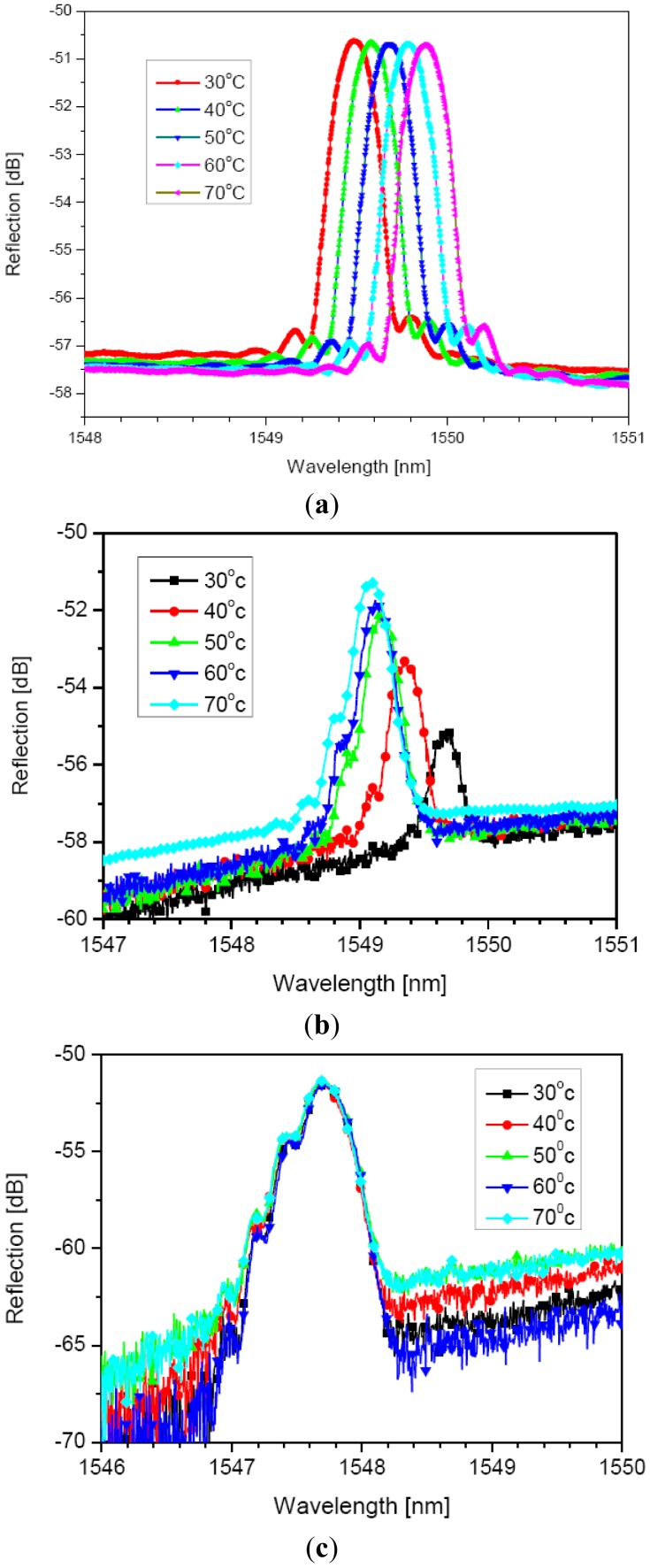
Spectral responses as a function of temperature of (**a**) non-cladding-etched FBG; (**b**) cladding-etched FBG in the liquid mixture of water (7%) and glycerin (93%); and (**c**) cladding-etched FBG in the liquid mixture of water (50%) and glycerin (50%).

As shown in Figure 4, during the cladding etching process, the effective refractive index of the fiber core decreases due to the evanescent wave coupling with the HF solution; thus, the Bragg wavelength of 1,550 nm shifts to the shorter wavelength of 1,547.4 nm, from which the remaining-cladding thickness can be derived, as seen in Figure 2. The remaining-cladding thickness was estimated to 0.3 μm. Figure 5 shows the reflection spectra of the cladding-etched FBG with the estimated remaining-cladding thickness of 0.3 μm as a function of temperature when two kinds of liquid mixtures were used: water (7%) and glycerin (93%), and water (50%) and glycerin (50%). These are compared with the reflection spectra of a conventional non-cladding-etched FBG.

Since the refractive indices of the liquid mixtures are dependent on the water and glycerin mixing ratio, the refractive indices of various weigh concentrations of water and glycerin were measured by using a commercial prism-coupling instrument.

The weigh concentration of water (7%) and glycerin (93%) was found to have a refractive index of 1.444, which is the same as that of fiber cladding, and that of water (50%) and glycerin (50%) was measured at 1.385.

The cladding-etched FBG was immersed in a metal container on a hot plate filled with the liquid mixtures of water and glycerin, and the temperature of the liquid mixtures was varied from 30 °C to 70 °C in a controlled manner using a hot plate (MST basic 2987000, IKA).

A conventional standard FBG has a well-known temperature sensitivity of 0.01 nm/°C; hence, the non-cladding-etched FBG in Figure 5(a) undergoes a Bragg wavelength of 1,549.5 nm shift to the longer wavelength of 1,549.9 nm when the temperature increases from 30 °C to 70 °C. As shown in Figure 5(b), the Bragg wavelength of 1,549.7 nm of the cladding-etched FBG with the liquid mixture of water (3%) and glycerin (93%) was shifted to a shorter wavelength, which shows a negative thermo-optic effect of the liquid mixture opposite to the positive thermo-optic effect of the standard FBG in Figure 5(a). With the greater thermo-optic effect of the liquid mixture than that of the silica FBG, the Bragg wavelength passed the central wavelength of 1,549.7 nm and shifted to a shorter wavelength. In Figure 5(c), in the liquid mixture of water (50%) and glycerin (50%) with the measured thermo-optic coefficient of −5 × 10^−4^ °C^−1^, the Bragg wavelengths of the proposed FBG were almost the same at 1,547.7 nm in the temperature range from 30 °C to 70 °C. Using this result, the Bragg wavelength shift dependent on the surrounding temperature can be avoided by the liquid mixture with a negative thermo-optic coefficient.

Experimentally we found out that the sharpness of the reflection spectrum of FBG degrades after wet etching. We inferred that non uniform thickness of fiber cladding after etching results in broadening of the reflection spectrum. Since etching speed of the silica fiber depends not only on the concentration of HF in etchant solution but also on the temperature of the liquid, non-homogeneous distribution in HF solution of the two physical parameters affecting etching speed may cause non uniform surface on etched fiber cladding surface. Our future research is to develop the etching technique to keep reflection spectrum after wet etching.

## 3. Conclusions

We have studied the temperature insensitivity of the cladding-etched FBG fiber immersed in a liquid mixture with a negative thermo-optic coefficient. The obtained results demonstrate that when a cladding-etched FBG fiber is coated with properly selected liquid mixtures with negative thermo-optic coefficients, the thermal-dependency of the etched FBG fiber can be effectively compensated.
